# Genetic Transformation of a *C. trachomatis* Ocular Isolate With the Functional Tryptophan Synthase Operon Confers an Indole-Rescuable Phenotype

**DOI:** 10.3389/fcimb.2018.00434

**Published:** 2018-12-14

**Authors:** Colette Elizabeth O'Neill, Rachel Jane Skilton, Sarah Ann Pearson, Simone Filardo, Patiyan Andersson, Ian Nicholas Clarke

**Affiliations:** ^1^Molecular Microbiology Group, Department of Clinical and Experimental Science, Southampton General Hospital, University Medical School, Southampton, United Kingdom; ^2^Section of Microbiology, Department of Public Health and Infectious Diseases, Sapienza University, Rome, Italy; ^3^Global and Tropical Health Division, Menzies School of Health Research, Charles Darwin University, Darwin, NT, Australia

**Keywords:** *Chlamydia trachomatis*, trachoma, interferon gamma, genetic complementation, transformation, tropism, tryptophan, plasmid

## Abstract

*Chlamydia trachomatis* is the leading cause of preventable blindness and the most common bacterial sexually transmitted infection. Different strains are associated with ocular or urogenital infections, and a proposed mechanism that may explain this tissue tropism is the active tryptophan biosynthesis pathway encoded by the genomic *trpRBA* operon in urogenital strains. Here we describe genetic complementation studies that are essential to confirm the role of tryptophan synthase in the context of an ocular *C. trachomatis* genomic background. Ocular strain A2497 was transformed with the (urogenital) pSW2::GFP shuttle vector showing that there is no strain tropism barrier to this plasmid vector; moreover, transformation had no detrimental effect on the growth kinetics of A2497, which is important given the low transformation efficiency of *C. trachomatis*. A derivative of the pSW2::GFP vector was used to deliver the active tryptophan biosynthesis genes from a urogenital strain of *C. trachomatis* (Soton D1) to A2497 with the aim of complementing the truncated *trpA* gene common to most ocular strains. After confirmation of intact TrpA protein expression in the transformed A2497, the resulting transformants were cultivated in tryptophan-depleted medium with and without indole or tryptophan, showing that complementation of the truncated *trpA* gene by the intact and functional urogenital *trpRBA* operon was sufficient to bestow an indole rescuable phenotype upon A2497. This study proves that pSW2::GFP derived vectors do not conform to the cross-strain transformation barrier reported for other chlamydia shuttle vectors, suggesting these as a universal vector for transformation of all *C. trachomatis* strains. This vector promiscuity enabled us to test the indole rescue hypothesis by transforming ocular strain A2497 with the functional urogenital *trpRBA* operon, which complemented the non-functional tryptophan synthase. These data confirm that the *trpRBA* operon is necessary and sufficient for chlamydia to survive in tryptophan-limited environments such as the female urogenital tract.

## Introduction

*Chlamydia trachomatis* is an obligate intracellular bacterium that is a major threat to human health. The species is divided into two biovars: trachoma and Lymphogranuloma venereum (LGV). This study focuses on the trachoma strains, which infect epithelial cells to cause two distinct diseases, namely the eye infection “trachoma” and the sexually transmitted infection “chlamydia.” The biological divide between ocular- and urogenital-associated strains is reflected by a clear phylogenetic separation, which occurred thousands of years ago (Hadfield et al., 2017). Whole genome sequencing has revealed that the trachoma biovar is divided into two distinct clades, T1 and T2 (Harris et al., 2012). T1 contains the common STI strains, whereas T2 contains two subclades comprising of the rarer STI strains and the ocular strains. The nesting of ocular strains within the T2 clade suggests that ocular *C. trachomatis* evolved from one common, urogenital ancestor (Harris et al., 2012).

Trachoma is prevalent in developing countries (in particular North Africa, India and South East Asia) where it is the leading infective cause of preventable blindness. Around 7.6 million people are currently affected, mainly in those areas prone to crowded living conditions, poor sanitation and limited access to medical services. Repeated exposure to the infection results in the accumulation of granular scar tissue on the conjunctiva leading to trichiasis, an inversion of the eyelids. This is an extremely painful condition, as with each blink the eyelashes scratch the eye surface leading to corneal opacity and, ultimately, blindness.

Trachoma was eliminated from Europe and North America in the early 1900s, largely due to improved sanitation and living conditions. Now, *C. trachomatis* is exclusively associated with the sexually transmitted infection (STI) chlamydia in these continents. This is the most common bacterial STI with around 131 million new infections reported yearly (Newman et al., 2015). Infection of the epithelial cells lining the cervix during the normal course of infection is frequently asymptomatic (around 70% in women), and thus may go undetected. Left untreated, the infection can ascend to the upper genital tract and cause serious and distressing long-term complications in women, including pelvic inflammatory disease (PID), ectopic pregnancy and infertility.

Despite this diversity of disease manifestations, the *C. trachomatis* genome is highly conserved; only 31 variable genes were identified by microarray analysis among the three biological tropisms (urogenital, ocular and LGV), 14 of which correlated to ocular tropism (Brunelle et al., 2004). *C. trachomatis* strains are traditionally divided by their MOMP (major outer membrane protein) serotype; in the majority of cases serovars A-C cause trachoma and strains D-K cause chlamydia. However, there is no known functional association between MOMP and tissue tropism; rather variation is thought to result from the antigenicity of MOMP and resulting immune selection (Brunelle and Sensabaugh, 2006) and recombination of the *ompA* gene has been observed between urogenital, ocular and LGV isolates (Harris et al., 2012), suggesting an alternative pathway(s) is likely to be responsible for chlamydial tissue tropism. A number of other genes have been implicated as having a role in tissue tropism, including *TarP* (translocated actin recruiting phosphoprotein) (Lutter et al., 2010) and six members of the polymorphic membrane protein family (Gomes et al., 2006). However, the most convincing evidence comes from phylogenetic studies that suggest the tryptophan biosynthesis operon as the defining feature of chlamydial tissue tropism (Shaw et al., 2000; Fehlner-Gardiner et al., 2002; Caldwell et al., 2003; Wood et al., 2003; Aiyar et al., 2014).

Although *C. trachomatis* obtains most of its nutrients parasitically from the host cell, in 1983 it was noticed that urogenital (but not ocular) strains of *C. trachomatis* were capable of growing *in vitro* in the absence of tryptophan (presumably in the presence of indole) (Allan and Pearce, 1983). This was surprising as *C. trachomatis* has a very small genome (1.04 Mb) consistent with its intracellular lifestyle: many biosynthetic pathways have been lost through the process of genome reduction (Horn et al., 2004; Read et al., 2013). However, three genes of the tryptophan biosynthesis pathway, *trpRBA*, have been retained (Stephens et al., 1998). These are located in the so-called “plasticity zone” of the *C. trachomatis* genome (Read et al., 2000), a region of around 50 Kb in length that accounts for the majority of sequence variation among *C. trachomatis* strains (Brunelle et al., 2004; Carlson et al., 2004; Thomson et al., 2008). In urogenital strains of *C. trachomatis, trpA* and *trpB* genes encode the functional units of the tryptophan synthase enzyme, which synthesises tryptophan from exogenous indole in response to tryptophan limitation, regulated by the tryptophan synthase repressor, TrpR (Fehlner-Gardiner et al., 2002; Wood et al., 2003; Akers and Tan, 2006). In contrast, classical trachoma strains have a single nucleotide deletion at position 533 of *trpA* causing early truncation of the TrpA protein (Shaw et al., 2000; Fehlner-Gardiner et al., 2002; Carlson et al., 2004). In *E. coli* TrpA is responsible for the conversion of IGP to indole, but this function has been lost in *C. trachomatis*, even where the intact *trpA* gene is present (Fehlner-Gardiner et al., 2002). The TrpB protein catalyses conversion of indole to tryptophan and thus proper functioning of tryptophan synthase is dependent on the expression of an intact TrpB protein (Miles, 1991, 1995, 2001). This was confirmed in *C. trachomatis* by chemical mutagenesis of *trpB* (Kari et al., 2011a). In *E. coli*, TrpB can function independently of TrpA, albeit at a lower level (Crawford and Yanofsky, 1958; Lane and Kirschner, 1983), whereas in *C. trachomatis* TrpA is absolutely required in addition to TrpB to produce a functional tryptophan synthase enzyme (Fehlner-Gardiner et al., 2002). Therefore, the observed truncation of ocular *C. trachomatis* TrpA eliminates the activity of tryptophan synthase (Fehlner-Gardiner et al., 2002). The largest genome survey of trachoma strains to date has confirmed that the majority of African isolates have this same truncating mutation (Last et al., 2018) and similar findings in the Pacific Islands suggest that this is a worldwide observation (Butcher et al., 2016). However, a recent publication documented a number of unusual ocular isolates from remote Aboriginal communities that appear to be urogenital-ocular hybrids, with ocular-associated *ompA* (genotypes B, Ba and C) and *pmpEFGH* genes, but, surprisingly, an intact (urogenital-type) *trpA* gene (Andersson et al., 2016). These strains form a separate lineage to the classical trachoma strains and may suggest a relatively recent adaptation of urogenital chlamydia to the eye in these communities, in stark contrast to the ancient ocular *C. trachomatis* addressed in the current study.

The retention of an operational *trpRBA* operon by urogenital *C. trachomatis* strains has been explained by the production of IFN-γ by host T cells and NK cells in the female urogenital tract (Fehlner-Gardiner et al., 2002). IFN-γ initiates the indoleamine 2,3-dioxygenase (IDO-1) cascade, catalysing the breakdown of intracellular stores of tryptophan (Taylor and Feng, 1991; Boehm et al., 1997). Tryptophan starvation results in *C. trachomatis* entering a quiescent and non-infective state, known as persistence (Beatty et al., 1993; Thomas et al., 1993). This is reversible, and infectivity can be rescued either upon the restoration of tryptophan availability, or by provision of indole by co-colonising microorganisms in the female urogenital tract (Morrison, 2003; Ziklo et al., 2016). Importantly, indole producers are absent from the eye. In the absence of indole, tryptophan synthase has another activity, the β-elimination reaction, which catalyses the breakdown of L-serine to produce ammonia (Kumagai and Miles, 1971). Ammonia is antimicrobial so this has been suggested as an explanation for the loss of *trpA* function in ocular strains of *C. trachomatis* (Aiyar et al., 2014) as in *E. coli* loss of *trpA* results in a 10-fold decrease in the B-elimination activity of TrpB (Ahmed et al., 1986). Together these studies form the basis of the indole-rescue hypothesis, offering an explanation of why urogenital but not ocular strains of *C. trachomatis* are able to evade the host IFN-γ response and thus survive in the female urogenital tract (Fehlner-Gardiner et al., 2002; Caldwell et al., 2003; Wood et al., 2003).

In this study, we have tested the hypothesis that the *trpRBA* operon of urogenital *C. trachomatis* is entirely responsible for the indole-rescue response using a shuttle vector derived from a urogenital strain plasmid (pSW2::GFP) to deliver the functional *trpRBA* operon to ocular *C. trachomatis* strain A2497. In doing so we prove that pSW2::GFP does not conform to the cross-strain transformation barrier reported elsewhere, identifying it as a universal vector appropriate for transformation of any strain of *C. trachomatis* and, critically, we compared the tryptophan-synthesis capability of the transformed ocular strain to its wild type counterpart to prove the indole rescue hypothesis.

## Methods

### Strains and plasmids

#### Plasmids

**Figure 1:**
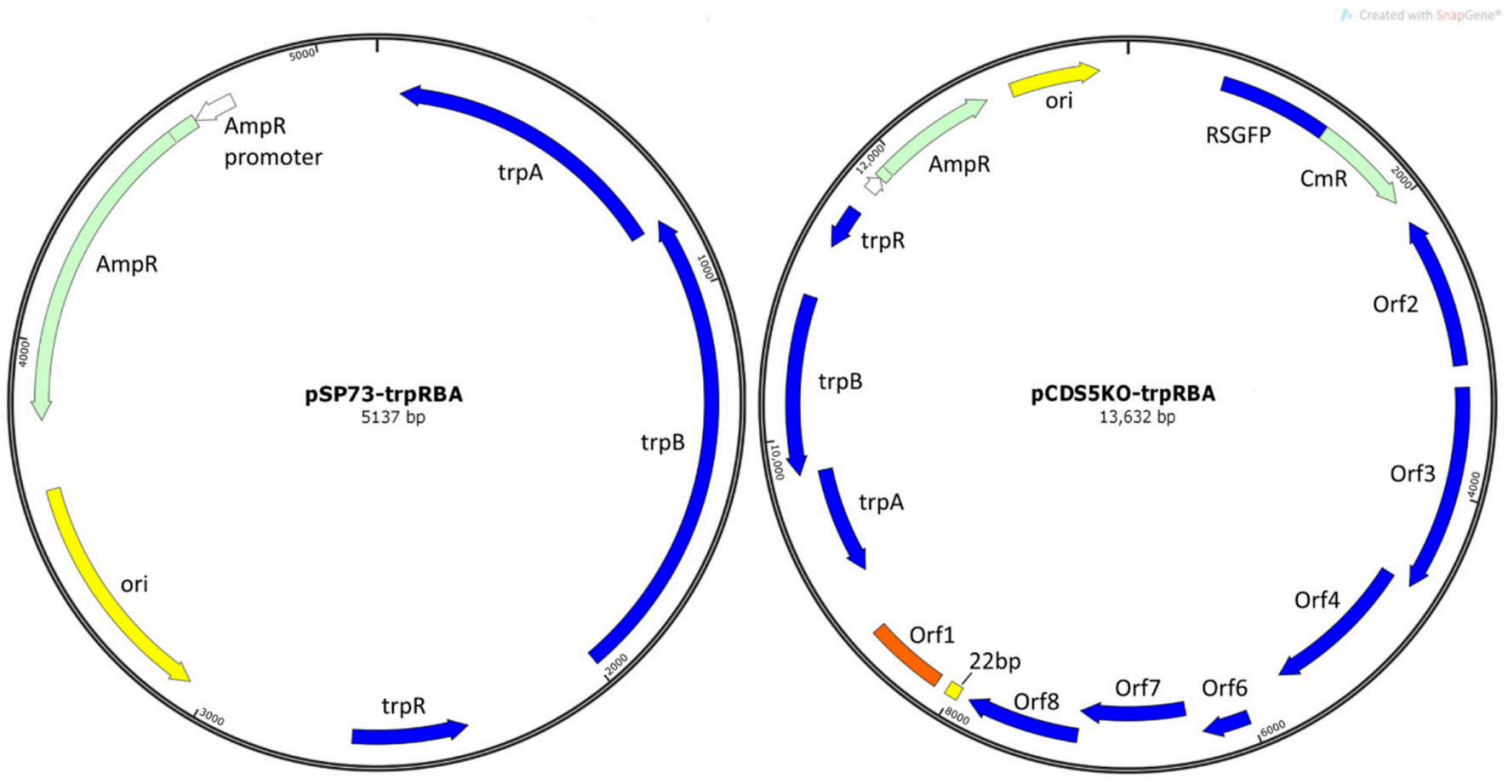
Plasmid maps of vectors constructed in this study. **(A)** *E. coli* vector pSP73::trpRBA and **(B)** shuttle vector pCDS5KO::trpRBA. *trpA, trpB*, and *trpR* indicate the genes of the tryptophan biosynthesis pathway, from Soton D1. Ori is the *E. coli* origin of replication. AmpR is the gene for ampicillin/penicillin resistance. CmR is the chloramphenicol acetyl-transferase gene for chloramphenicol resistance; RSGFP is the green fluorescence protein, and each of the chlamydial plasmid genes CDS1-8 are shown (except CDS5, which is deleted in pCDS5KO).

Plasmids pSW2::GFP and pCDS5KO were constructed during previous studies (Wang et al., 2011a and Wang et al., 2013b respectively). pSP73 is a standard *E. coli* commercial cloning vector (Promega). pSP73::trpRBA and pCDS5KO::trpRBA were constructed during this study (Figure 1).

#### E. coli

*E. coli* strain DH5α (Hanahan, 1985; Grant et al., 1990) was used for construction of plasmid vectors using standard CaCl_2_ treatment to render the cells competent (Brown, 1995). *E. coli* strain BW25113 was obtained from the Coli Genetic Stock Centre (Baba et al., 2006) and used as a positive control for tryptophan synthesis from indole. *E. coli* strain K12ΔHIΔtrp (Bernard et al., 1979) was used as the recipient strain for pCDS5KO::trpRBA to test the tryptophan synthesis capability conferred by this plasmid, as this strain is a complete tryptophan biosynthesis pathway knockout. This strain also has a requirement for biotin due to a Δbio-uvrB mutation (Bernard et al., 1979). GM2163 is a Dam-/Dcm- strain, and was used to prepare unmethylated DNA for transformation of *C. trachomatis* (NEB).

#### C. trachomatis

*Chlamydia trachomatis* A2497WT was isolated from a 2.5-year-old with intense active trachoma in Tanzania in 2001 (Kari et al., 2008). *C. trachomatis* Soton D1 is a typical D serovar endocervical isolate, which was isolated from Southampton in 2009 (Wang et al., 2011b). Although referred to as EBs throughout this publication, chlamydia inocula are not purified and will therefore contain some RBs as well. However, as these were quantified by titration, the number of inclusion forming units (IFUml^−1^) applies to EBs alone. McCoy (mouse fibroblast) and HEp2 (HeLa-derivative human cervix carcinoma) cells were obtained from the National Collection of Type Cultures (NCTC) and routinely propagated in DMEM (+Glutamax -Pyruvate) medium (Gibco) supplemented with 10% FCS.

*C. trachomatis* strains were grown routinely in McCoy cells in DMEM-complete with 1 μgml^−1^ cycloheximide and 20 μgml^−1^ gentamicin (referred to as DMEM-complete). After inoculation, flasks or tissue culture trays were centrifuged for 30 min at 754 *g* and incubated at 37°C/5% CO_2_ for 48 h.

During tryptophan phenotype experiments, chlamydia strains were grown in HEp2 cells in custom-made tryptophan-free medium (referred to as DMEM-Trp) (Athena Enzyme Systems, United States); indole or tryptophan was added as necessary. The chemical composition of DMEM-Trp is the same as DMEM-complete, but does not contain any tryptophan. This was supplemented with 10% dialysed FCS (ThermoFisher).

All chlamydia and cell lines were tested routinely for Mycoplasma using the LookOut® Mycoplasma PCR Detection Kit (Sigma) and verified free from contamination.

### Construction of pSP73::trpRBA and pCDS5KO::trpRBA

Basic cloning vector pSP73 was digested with SalI restriction enzyme (Promega) then dephosphorylated using Antarctic Phosphatase (NEB). Whole genomic DNA was purified from *C. trachomatis* strain Soton D1 using the Wizard Genomic DNA purification kit (Promega), and the *trpRBA* operon (including the native promoter sequence) was PCR amplified from Soton D1 using Phusion Flash High Fidelity PCR Master Mix (ThermoFisher Scientific) with primers Trp_F_SalI (5′-aaaaaagtcgaccgtgatccttattcctagaggggagaattc-3′) and Trp_R_SalI (5′-aaaaaagtcgacttatccaggaataactgtttgtgcaagtgc-3′), including SalI restriction sites (underlined). The amplicon incorporates the natural promoter sequence for the *trpRBA* operon. The PCR conditions were 1 min initial denaturation at 98°C; then 40 cycles of 98°C denaturation for 1 s, 60°C annealing for 5 s and extension at 72°C for 40 s; followed by a final extension step of 72°C for 1 min. The *trpRBA* amplicons were gel purified using the Wizard® SV Gel and PCR Clean-Up System (Promega) and digested with SalI restriction enzyme as above. Plasmid pSP73 and the *trpRBA* amplicon were ligated using T4 DNA ligase (Promega), using a 1:3 *trpRBA* to pSP73 ratio, to give pSP73::trpRBA (Figure 1A). The ligation was transformed into chemically competent DH5α cells. 50 μgml^−1^ ampicillin was used to select transformants. Colonies were screened for the presence of the *trpRBA* operon by PCR, using primers Trp_F_SalI and Trp_R_SalI. Once verified, plasmid DNA was extracted from overnight broth cultures of these pSP73::trpRBA positive colonies using the Wizard® Plus SV Minipreps DNA Purification System (Promega). Following confirmation of the activity of *trpRBA*, purified pSP73::trpRBA DNA was digested with SmaI restriction enzyme (NEB), which was heat inactivated, then with PvuI and PvuII (double digest, using NEB enzymes and buffer 3.1) to release the *trpRBA* fragment and break up the plasmid backbone. The 2.7 Kb *trpRBA* operon was isolated by gel extraction using the Wizard® SV Gel and PCR Clean-Up System (Promega). The pCDS5KO shuttle vector (Wang et al., 2013b) was digested with ZraI (NEB). After dephosphorylation (as above), pCDS5KO was ligated with *trpRBA* (1:7 plasmid to insert ratio) and incubated overnight at 16°C, and heat inactivated at 70°C for 10 min prior to transformation into chemically competent DH5α *E. coli*, as described above. Clones were selected with ampicillin (50 μgml^−1^), and carriage of the *trpRBA* operon by the resulting plasmid (pCDS5KO::trpRBA; Figure 1B) was verified by PCR, as described above. *TrpRBA*-positive colonies were grown in LB broth (with 50 μgml^−1^ ampicillin) overnight, and plasmid DNA was extracted as described above. Finally, *E. coli* strain GM2163 was transformed with pCDS5KO::trpRBA or pSW2::GFP and concentrated plasmid DNA for chlamydia transformations was isolated from this strain using the Wizard® plus Midiprep kit following the manufacturer's instructions (Promega).

### Transformation of A2497WT and A2497P- With pSW2::GPF or pCDS5KO::trpRBA

A2497WT and A2497P- were transformed with 6 μg of pSW2::GFP (Wang et al., 2011a) or pCDS5KO::trpRBA similarly to previously described methods (Wang et al., 2013a), with the exceptions: (1) 2 × 10^7^ A2497 EBs were transformed and (2) chlamydia EBs were pelleted and re-suspended in 150 μl of CaCl_2_ buffer prior to mixing with 6 μg of plasmid DNA (10 μl of 600 ngμl^−1^ DNA in 90 μl of CaCl_2_ buffer). Once transformants were recovered, whole genomic DNA from A2497/pSW2::GFP was extracted and cloned into *E. coli* DH5α to investigate whether any recombination had occurred between native plasmid and the shuttle vector. Ampicillin-resistant colonies were selected and plasmid DNA was then extracted from individual colonies. These were digested using HindIII and the fragment sizes compared. As these all appeared identical, a representative DNA extract was subjected to whole plasmid sequencing (CCIB DNA core, Massachusetts General Hospital, United States). The resulting sequence was interrogated using SeqBuilder™ (version 12.3.1, DNASTAR).

### Plasmid Curing of A2497/pSW2::GFP

Plasmid curing of transformed A2497/pSW2::GFP was performed using novobiocin, as previously described (O'Connell and Nicks, 2006), at a novobiocin concentration of 62.5 μgml^−1^. A total of 10 rounds of limiting dilutions in 96-well tissue culture trays were used to isolate an individual plasmid-free inclusion to ensure clonality, prior to expanding in cell culture.

### One Step Growth Curves

#### Infectivity Assays

12-well tissue culture trays were seeded with McCoy cells the day before the start of the experiment. Once the monolayer was near-confluent, the appropriate amount of *C. trachomatis* A2497WT, A2497P-, or A2497P-/pSW2::GFP inoculum was added to give an MOI ≤ 1. Infection proceeded as described above. Samples were collected at 0, 8, and then every 4 h until 44 h post infection, after which the experiment ended as cell lysis had begun, signifying the end of the reproductive cycle. At each time point, cells were scraped into the supernatant with a 1 ml pipette tip and transferred to a bijou tube containing glass beads and 1 ml of 4SP buffer. These were frozen at −80°C. To enumerate the infectivity, each sample was thawed, vortexed for 1 min to disrupt cells, and 100 μl of inoculum was transferred into a 96-well tray (in duplicate). Ten-fold dilution series were made in DMEM-complete. The dilution series were then transferred to 96-well trays pre-seeded with McCoy cells. These were infected as described above. The trays were incubated at 37°C/5% CO_2_ for 44 h. Cells were then fixed with methanol and incubated for 20 min at −20°C. After three washes in PBS, cells were incubated overnight at 4°C with a 1:1,000 dilution (in PBS) of the mouse monoclonal Chlamydia genus-specific anti-LPS antibody MAb29 (Chlamydia Biobank, CT601. RRID:AB_2721933) (Skilton et al., 2007), before subjecting to the X-Gal staining procedure, as previously described (Skilton et al., 2007). Stained inclusions were then counted in the duplicate wells at an appropriate dilution and IFUml^−1^ was calculated. Growth rates during the exponential phase were calculated using N_t_ = N_o_(1+r)^t^ and compared to each other using the Student's *t*-test; additionally, titres were calculated for each time point and a 2-way ANOVA was performed using GraphPad Prism (version 7.03) to compare the overall yields throughout the experiment.

#### Confocal Microscopy

12-well trays containing glass microscope coverslips were seeded with McCoy cells the day before the start of the experiment. Cells were infected with the appropriate amount of *C. trachomatis* A2497WT, A2497P-, or A2497P-/pSW2::GFP inoculum to give an MOI of 1. The 12 well trays were centrifuged and incubated at 37°C/5% CO_2_ as described above. At each time point, cells were fixed using 4% paraformaldehyde for 15 min. Cells were washed in PBS and permeabilised in saponin buffer (0.1% saponin, 10% foetal calf serum, 0.1% sodium azide) for 1 h at 4°C. Monoclonal antibody MAb29 was added (in saponin buffer) at 1:1,000 dilution and cells were incubated for 1 h at room temperature. These were then washed three times in saponin buffer before the anti-mouse-Alexa-fluor 488 conjugate antibody (Fisher Scientific) was added at 1:200 dilution. This was again incubated for 1 h at room temperature, washed as before and counterstained with 1 μg/ml DAPI (Fisher Scientific) and Wheat Germ Agglutinin Alexa Fluor® 594 conjugate (Invitrogen), washed a final time in PBS and mounted onto slides with Mowiol mounting medium (Sigma Aldrich). Images were captured using a Leica TCP SP8 confocal microscope. Areas of inclusions were measured at each time point using ImageJ (version 1.51j8) and compared using a one-way ANOVA using GraphPad Prism (version 7.03).

### Expression Analysis of TrpA

To ascertain whether the intact TrpA protein was being expressed by the *trpA*-complemented A2497 strain, western blot analysis was performed. Hep2 cells were grown overnight in DMEM-Trp in 24 well trays and infected with *C. trachomatis* strains A2497WT, A2497/pCDS5KO::trpRBA or Soton D1 as described above. After centrifugation, the growth medium was exchanged for DMEM-Trp, DMEM-Trp plus indole (50 μM) or DMEM complete (all with cyclohexmide at 1 μgml^−1^). Trays were incubated at 37°C/5% CO_2_ for 48 h, after which time each well was harvested by scraping the infected cells into the medium, which was transferred to a 5 ml bijou tube containing glass beads, and cells were disrupted by vortexing for 1 min. Harvests were frozen in 1:1 4SP at −70°C. One millilitre of each harvest was concentrated by centrifugation at 14,000 × g for 2 min. The pellets were resuspended in 10 μl of PBS and boiled in the presence of β-mercaptoethanol for 5 min, prior to SDS-PAGE electrophoresis (using a 12% polyacrylamide gel). Anti-TrpA polyclonal antisera raised in rabbits against purified, poly-His tagged TrpA protein (originating from *C. trachomatis* Soton D1) was used to bind chlamydial TrpA protein, and goat anti-rabbit HRP conjugated secondary antibody was used to detect the bound anti-TrpA antibody. Western blot analysis was performed as previously described (Wang et al., 2011a). The antibodies were then stripped (by incubating the membrane with stripping buffer (pH 6.7) containing 100 Mm β-mercaptoethanol, 2% SDS, and 62.5 mM Tris at 50°C for 30 min, followed by washing in PBS-T three times) and the membrane was re-probed with MAb29 to detect lipopolysaccharide (LPS) as an indicator of chlamydial growth yield.

### Testing of pCDS5KO::trpRBA Phenotype in *E. coli*

The indole-rescue phenotype conferred by pCDS5KO::trpRBA was confirmed by transforming the plasmid into *E. coli trpEDCBA* knockout mutant K12ΔH1Δtrp. Competent DH5α cells were transformed with 10 ng of pCDS5KO::trpRBA DNA and transformants were selected with ampicillin (50 μgml^−1^). Transformants were verified by PCR of the *trpRBA* operon using primers Trp_F_SalI and Trp_R_SalI, as detailed above. Transformants were sub-cultured onto M9 minimal medium plates (Sambrook et al., 1989) supplemented with glucose (0.4%), biotin (5 ngml^−1^) and either tryptophan (50 μgml^−1^) or indole (50 μM), or minimal medium without tryptophan or indole. Plates were incubated overnight at 37°C and examined for growth the next day.

### Indole-Rescue Phenotypes of A2497/pCDS5KO::trpRBA, A2497WT and Soton D1

A2497/pCDS5KO::trpRBA, A2497WT and the *trpRBA* operon donor strain Soton D1 were compared on their ability to grow in DMEM-Trp, with and without the addition of indole (50 μM) or tryptophan (20 μgml^−1^). 6-well trays were seeded with human epithelial HEp2 cells, in DMEM-Trp medium and also in DMEM-Trp with the addition of 5 ngml^−1^ IFN-γ as an extra measure to mop up any residual tryptophan. The cells were grown to confluence for 24 h prior to infecting with chlamydia to ensure intracellular pools of tryptophan had been used up. Wells were inoculated with chlamydia at an MOI of 1. These were centrifuged and the medium exchanged for (1) DMEM-Trp (2) DMEM-Trp plus indole and (3) DMEM-Trp plus tryptophan (all with cycloheximide at 1 μgml^−1^); these three conditions were repeated with the addition of IFN-γ. The trays were incubated for 48 h, and each well was harvested into 1 ml of 10% PBS, from which 10-fold dilution series were made. One hundred microlitre of each dilution was inoculated into a 96 well tray of confluent HEp2 cells in DMEM-complete plus cycloheximide and gentamicin (in triplicate). After centrifugation of the 96 well trays, these were incubated for 44 h at 37°C/5% CO_2_. The wells were then washed twice with 100 μl of PBS, and fixed by adding ice-cold methanol. Trays were then incubated at −20°C for 20 min, before washing twice with PBS. 100 μl of monoclonal antibody MAb29 was added at a dilution of 1:1,000 in PBS and incubated overnight at 4°C before subjecting to the X-Gal staining procedure, as previously described (Skilton et al., 2007).

## Results

### Transformation of Ocular *C. trachomatis* A2497 With Plasmid Vector pSW2::GFP

**Figure 2:**
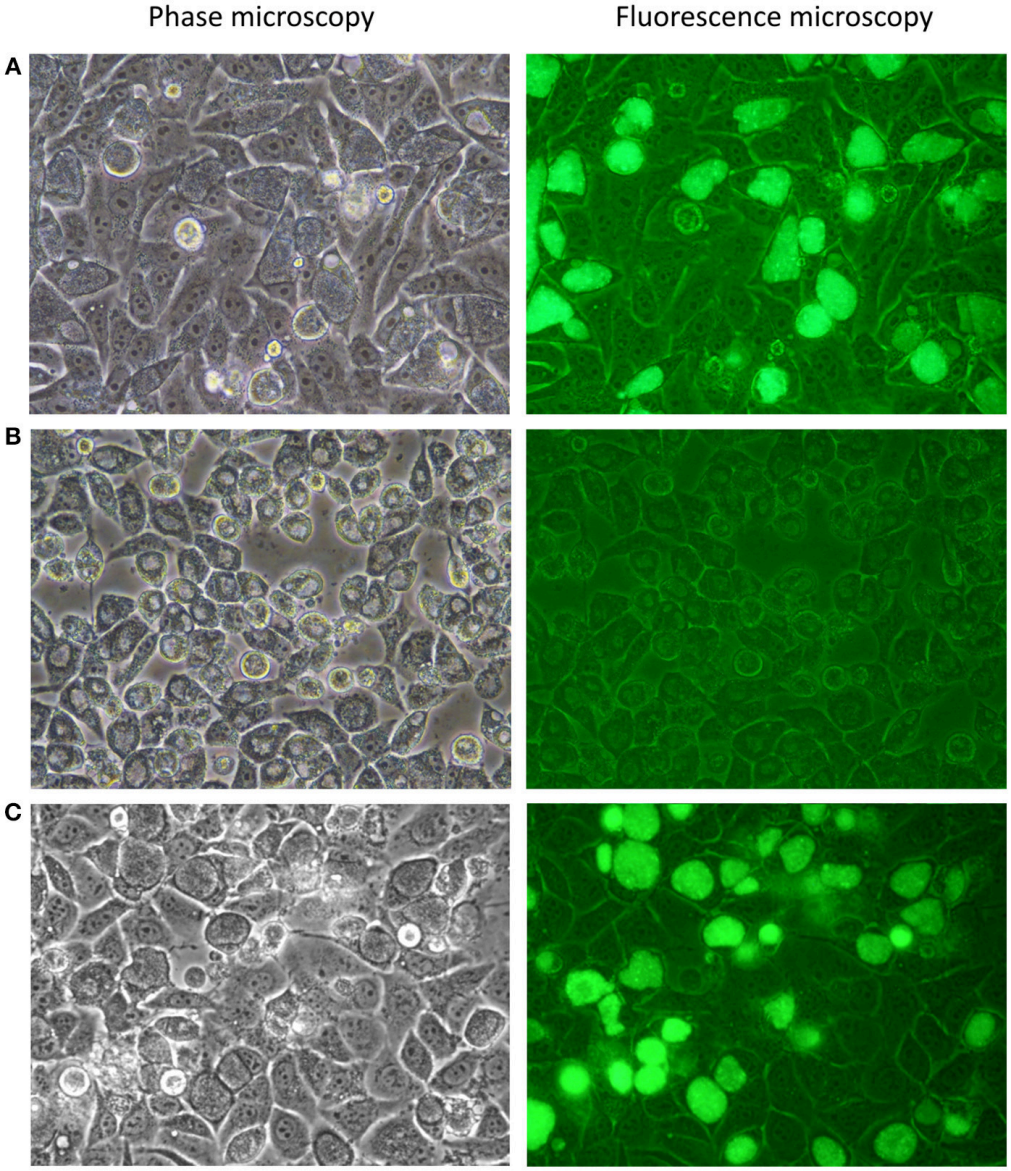
Photomicrographs of *C. trachomatis* A2497 strains visualised by confocal and fluorescence microscopy. **(A)** A2497WT transformed with pSW2::GFP; normal inclusion morphology and green fluorescence are evident, respectively. **(B)** Plasmid-cured A2497 (A2497P-). Plasmid-free inclusion morphology can be seen by confocal microscopy, typified by a dark border, and pale centre. There is no fluorescence under UV light due to the loss of the GFP gene, which is carried by the plasmid. **(C)** Restoration of normal inclusion morphology and green fluorescence in A2497P- transformed with the pSW2::GFP plasmid. All strains were grown without selection in the presence of cycloheximide (1 μgml^−1^) and gentamicin (20 μgml^−1^).

A recent study suggested there is a significant plasmid-based tropism barrier even within *C. trachomatis* biovars (Song et al., 2014). Transformation of *C. muridarum* with urogenital *C. trachomatis*-derived plasmid pSW2::GFP yielded recombinant plasmids identifying CDS2 as the inter-species tropism-determining feature of the plasmid (Wang et al., 2014). Therefore, our initial experimental design was to transform plasmid-bearing wild type *C. trachomatis* trachoma isolate A2497 (referred to henceforth as A2497WT) with shuttle vector pSW2::GFP. Transformants were recovered after two passages under penicillin selection. Transformants had normal inclusion morphology, were resistant to 10 Uml^−1^ penicillin and fluoresced green under UV light (Figure 2A). To ascertain whether pSW2::GFP had recombined with the native plasmid of A2497WT, whole-genomic DNA was extracted from the transformed A2497WT and cloned into *E. coli* DH5α. Sequencing of recovered pSW2::GFP plasmid showed that it was identical to the original vector as reported by Wang et al (Wang et al., 2011a). This is consistent with previous data showing that pSW2::GFP is able to transform LGV strain L2 (which is more distantly related to urogenital than trachoma strains) without the need for recombination with the endogenous plasmid (Wang et al., 2011a). These data proved that the pSW2::GFP plasmid could replicate in A2497 without recombination with the endogenous plasmid. The next step was to show that a plasmid-free recipient host could also be transformed with pSW2::GFP.

### Plasmid Curing and Transformation of *C. trachomatis* A2497P-

To complete the analysis of genome:plasmid compatibility between A2497 and pSW2::GFP, the A2497/pSW2::GFP transformant was cured of the shuttle vector using standard plasmid curing protocols (O'Connell and Nicks, 2006) so that it could subsequently be re-transformed. After treatment with 62.5 μgml^−1^ novobiocin, approximately 50% of A2497 inclusions had lost the plasmid after a single passage in novobiocin-free medium (as judged by observing the loss of green fluorescence under UV light, combined with the appearance of inclusions exhibiting the plasmid-free phenotype; Figure 2B). After six rounds of limiting dilutions, an individual well containing a single, plasmid-free inclusion was identified. The cells in this well were harvested and four rounds of limiting dilutions were performed to ensure clonality. This was then expanded in cell culture, resulting in a pure, clonal plasmid-cured *C. trachomatis* A2497 inoculum (A2497P-). *C. trachomatis* A2497P- has a distinct inclusion morphology with a dark border and pale centre, and does not fluoresce green under UV light (Figure 2B). Furthermore, end-point PCR amplification of a plasmid-specific target (using primers described by Pickett et al Pickett et al., 2005) showed that no residual plasmid DNA remained in the plasmid-free strain (data not presented).

Subsequent transformation of the plasmid-cured A2497P- with pSW2::GFP was successful and easily reproduced (n = 6), restored normal inclusion morphology and inclusions fluoresced green under UV light, as expected (Figure 2C). This confirmed the finding that recombination with the native plasmid is not a pre-requisite for cross-strain transformation with this vector.

### Growth Kinetics

**Figure 3:**
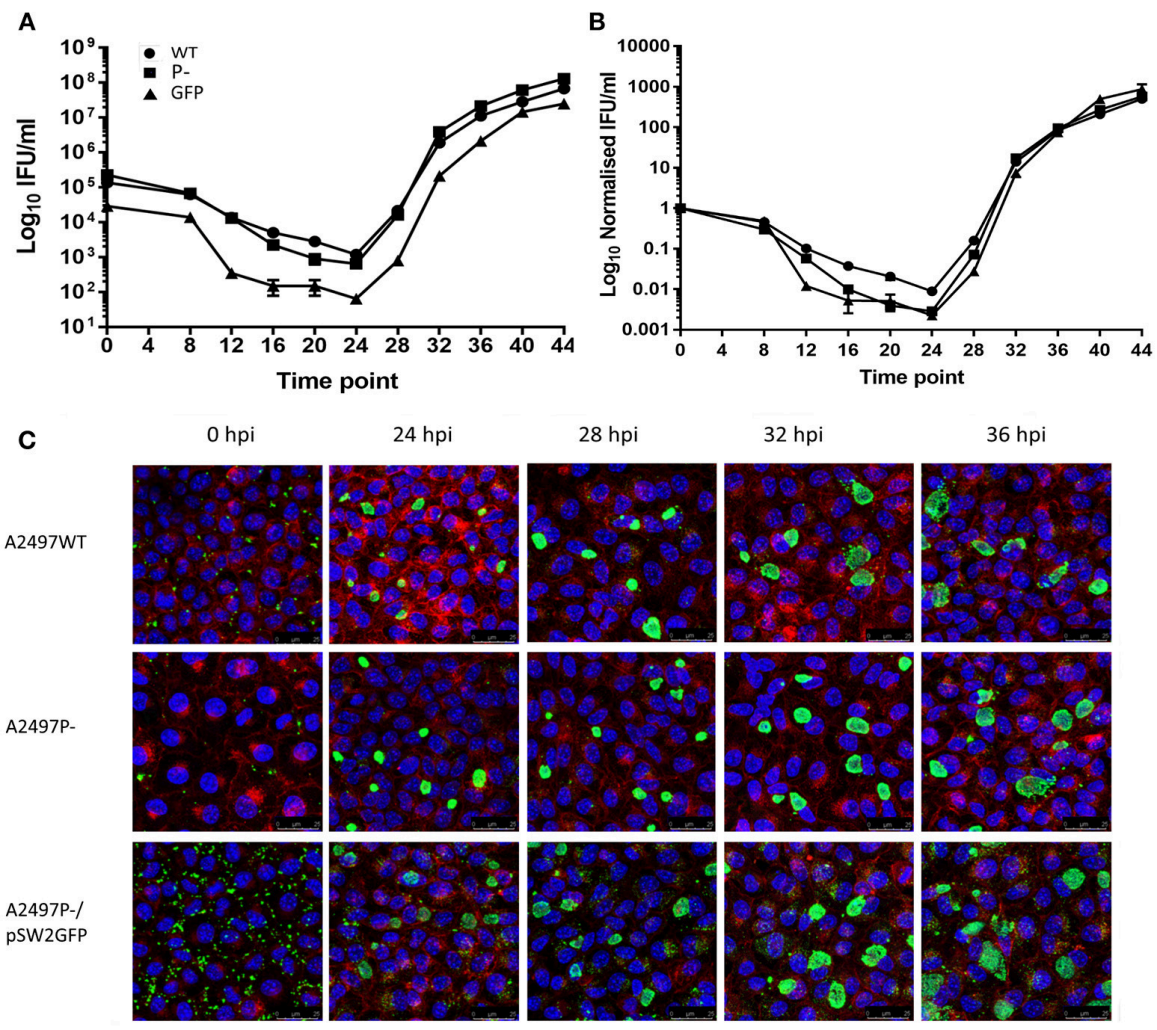
Growth curve analysis of *C. trachomatis* A2497 strains used in this study: wildtype (WT), plasmid cured (P-), and carrying the pSW2::GFP plasmid (GFP). **(A)** One-step infectivity assay; this is plotted on a log_10_ Y axis; **(B)** normalised infectivity assay. Here, the inclusion counts (IFUml-^1^) were normalised by dividing each value by the starting IFUml-^1^ to make all curves relative to 1 IFUml-^1^ for ease of comparison. **(C)** Confocal images of immunofluorescently stained inclusions at selected time points.

Shuttle vector pSW2::GFP is larger than the native plasmid (11.5 kb vs. 7.5Kb, respectively), expressing a chloramphenicol acetyltransferase (CAT) green fluorescent protein (GFP) fusion protein as well as β-lactamase, and thus may incur a fitness burden. To investigate the impact of carriage of this plasmid on chlamydial growth kinetics, one-step growth curves were performed to compare the transformed strain to those of the wild type parent strain and plasmid-cured derivative. Samples were taken every 4 h until cell lysis began at 44 h post infection. Each strain was titrated and the inclusion forming units per ml (IFUml^−1^) calculated at each time point (Figure 3A). Data were normalised to 1 for ease of comparison (Figure 3B). We found that the transformants did not have a reduced growth rate in comparison to either A2497WT or A2497P-, and the final yield was not significantly different, suggesting that transformation by pSW2::GFP does not incur any fitness burden *in vitro*. We also performed immunofluorescence staining on coverslips obtained at selected time points throughout the developmental cycle and measured the average inclusion sizes (Figure 3C and Supplementary Figure 2). There was no significant difference between the strains at any time point–the inclusions of transformed chlamydia were the same size as those for *C. trachomatis* A2497WT or A2497P- throughout the growth cycle.

### Construction of pCDS5KO::trpRBA and Testing of Indole Rescue Phenotype

The *trpRBA* operon (with the native promoter) from urogenital *C. trachomatis* strain Soton D1 was cloned into the pCDS5KO shuttle vector, producing pCDS5KO::trpRBA. pCDS5KO is a derivative of pSW2::GFP, with CDS5 deleted (Wang et al., 2013b). This was chosen to reduce the overall size of the final vector–the system suffers from a low efficiency of transformation so we felt this was a prudent step to improve our chances of recovering transformants. The ability of the *trpRBA* operon to synthesise tryptophan from indole was first confirmed in *E. coli* strain K12ΔH1Δtrp (a complete tryptophan synthesis operon deletion mutant; Bernard et al., 1979). Where K12ΔH1Δtrp could only grow on minimal medium supplemented with tryptophan, K12ΔH1Δtrp transformed with pCDS5KO::trpRBA was also able to grow on M9/indole, confirming the activity of *trpRBA* in this construct (Table 1). These data concurred with previous reports of chlamydial *trpRBA* functionality in other *E. coli* tryptophan auxotroph mutants (Fehlner-Gardiner et al., 2002).

**Table 1:** Indole rescue phenotypes of *E. coli* strain K12ΔHIΔtrp with and without complementation by vector pCDS5KO::trpRBA.

**Strain**	**Minimal medium M9**	**M9 + Tryptophan**	**M9 + Indole**
K12ΔHIΔtrp	−	+	−
K12ΔHIΔtrp/pCDS5KO::trpRBA	−	+	+

*E. coli strains were grown on minimal medium M9 agar plates, with and without supplementation by tryptophan or indole. After incubation for 24 h, growth was assessed visually and is indicated by +/− (growth/no growth). Both strains grew in the presence of tryptophan and neither grew in the absence of tryptophan or indole. Only K12ΔHIΔtrp/pCDS5KO::trpRBA was able to grow in the absence of tryptophan when supplemented with indole*.

Once the tryptophan biosynthesis function was confirmed in *E. coli* K12ΔHIΔtrp, pCDS5KO::trpRBA was used to transform ocular *C. trachomatis* strain A2497P-. Phase contrast microscopy revealed that inclusions had a normal morphology that was indistinguishable from A2497WT or A2497/pSW2::GFP inclusions under phase contrast (data not shown).

The *trpA* gene is truncated in most ocular strains of *C. trachomatis*. Complementation of this by the *trpRBA* operon of a urogenital strain was expected to result in restoration of tryptophan synthesis capability through the expression of an intact TrpA protein. Expression of TrpA was assessed using western blot analysis, using a polyclonal anti-serum raised against purified, His-tagged TrpA protein. No discernible protein was seen for A2497WT due to truncation of TrpA–this may be due to the loss of epitopes required for TrpA anti-serum reactivity. Soton D1, on the other hand, expressed the full-length TrpA protein (Supplementary Figure 1). The complemented A2497/pCDS5KO::trpRBA strain expressed a protein of the same size as that seen for Soton D1, proving that TrpA is expressed from the pCDS5KO::trpRBA plasmid in A2497. When grown in the presence of excessive tryptophan, expression of TrpA was noticeably weaker than when grown in the absence of tryptophan, as can be seen by comparing the band intensities to the loading control (LPS, as detected by monoclonal antibody MAb29). This proves that the *trpA* gene present on the shuttle vector is expressing TrpA in the transformed A2497 strain in response to tryptophan limitation, and suggests that in the presence of excess tryptophan expression of TrpA is being down-regulated by the TrpR protein, as expected.

The indole-rescue phenotype of A2497/pCDS5KO::trpRBA was assessed using artificially tryptophan-depleted medium supplemented with either indole or tryptophan (with and without the addition of IFN-γ), in comparison to *trpRBA* donor Soton D1 (positive control) and *trpRBA* recipient strain A2497WT (negative control). *C. trachomatis* strains were grown in HEp2 cells that had been deprived of tryptophan for 24 h prior to infection, with and without the addition of IFN-γ. After infection, the cell culture medium was exchanged for DMEM-Trp (tryptophan-depleted media), DMEM-Trp+Indole or DMEM-Trp+Tryptophan, and incubated for 48 h (all with and without the addition of IFN-γ). *C. trachomatis* was then harvested and titrated in HEp2 cells that had been grown overnight in complete DMEM medium in 96 well trays. After 44 h these were fixed and stained using the X-Gal staining procedure.

**Figure 4:**
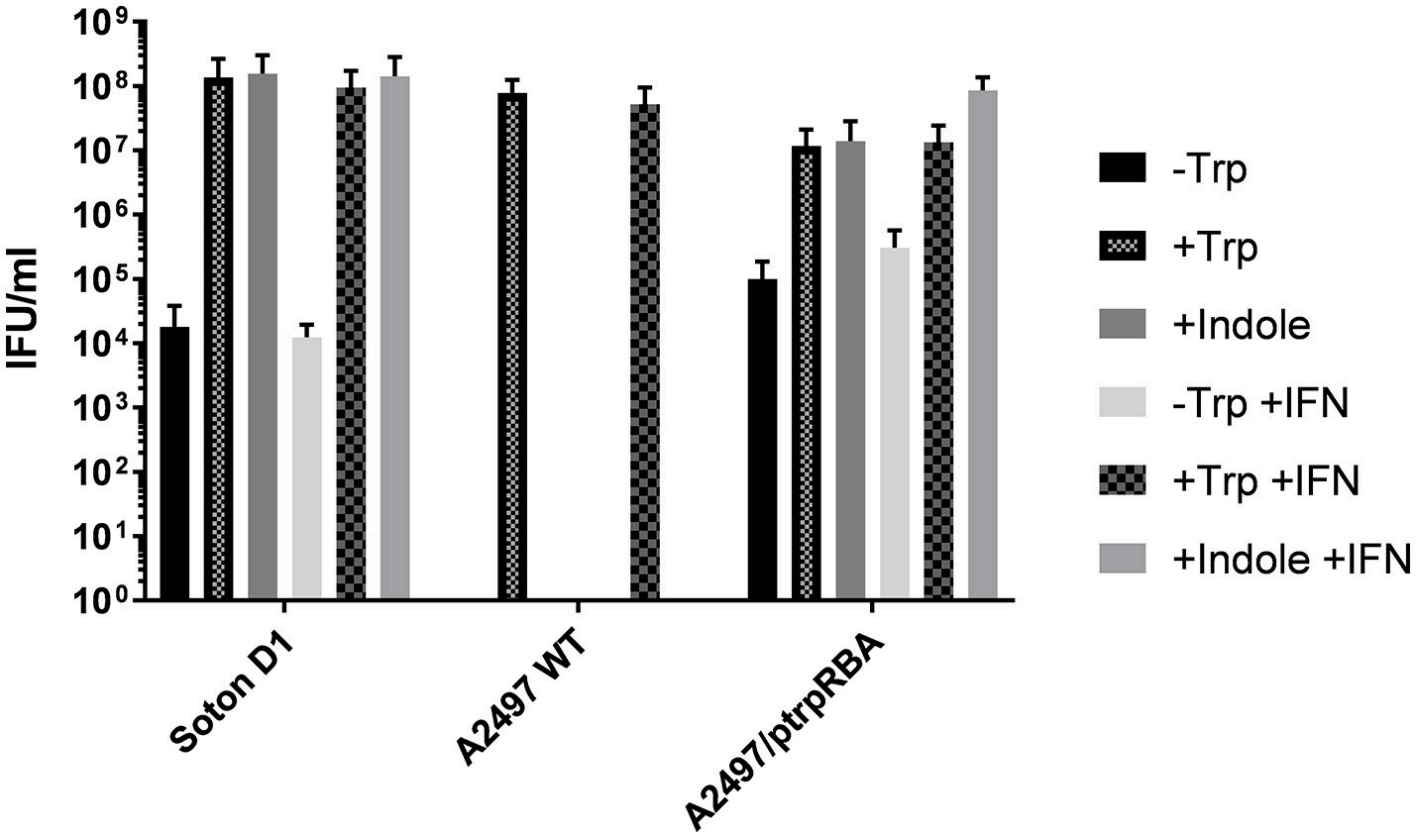
Indole rescue phenotypes of Soton D1, A2497WT, and A2497/pCDS5KO::trpRBA. Each strain was grown in DMEM-Trp medium (-Trp) and supplemented with tryptophan (+Trp) or indole (+Indole), with and without the addition of IFN-γ. After 48 h incubation, these were harvested and titrated in DMEM-complete in 96 well trays, in triplicate. Trays were fixed after 44 h and stained using the X-Gal staining procedure.

Infectivity of A2497WT was eliminated when grown in DMEM-Trp or DMEM-Trp+indole, but high titres were obtained upon supplementing with tryptophan, as expected (Figure 4). The positive control strain Soton D1 retained its infectivity when grown in DMEM-Trp+indole and DMEM-Trp+tryptophan, but infectivity was reduced when grown in DMEM-Trp. Similarly, growth of A2497WT/pCDS5KO::trpRBA was reduced in DMEM-Trp, and high titres were recovered in both DMEM-Trp+tryptophan and DMEM-trp+indole, proving the activity of the urogenital *trpRBA* operon in isolation from other urogenital strain chromosomal loci. The presence of inclusions (in low numbers) in the -Trp experiment for all strains except A2497WT may be explained by carry-over of inoculum from the first stage of the experiment, or to the presence of residual tryptophan in the cells or inoculum. The results for the artificially tryptophan-depleted medium and that treated with IFN-γ were highly similar, suggesting that these methods are equally appropriate for the assessment of indole-rescue phenotypes.

These data confirm that complementation by the pCDS5KO::trpRBA plasmid bestows an indole-rescuable phenotype on ocular strain A2497WT.

## Discussion

The underlying mechanisms that divide *C. trachomatis* strains between their respective ocular and urogenital niches have been the subject of scientific scrutiny for 20 years (Stothard et al., 1998). A body of evidence based on genome phylogeny supports the hypothesis that retention of an active *trpRBA* operon by urogenital (but not ocular) strains is the key determinant of chlamydial tropism (Shaw et al., 2000; Fehlner-Gardiner et al., 2002; Caldwell et al., 2003; Akers and Tan, 2006; Aiyar et al., 2014). The role of the *trpRBA* operon in *C. trachomatis* was first deduced by comparing urogenital and ocular strains on their ability to synthesise tryptophan from indole in response to tryptophan limitation (Fehlner-Gardiner et al., 2002; Caldwell et al., 2003). However, in genetically tractable bacteria the key experiment to prove gene function is by complementation. For chlamydia this technology has only recently been developed (Wang et al., 2011a) and, so far, only one study has applied this to the genes of the *trpRBA* operon (Mueller et al., 2016). The authors of that study used genetic complementation of *trpA* as proof-of-principle for the function of a newly developed suicide vector and a new gene editing method, fluorescence-reported allelic exchange mutagenesis (FRAEM). However, complementation was performed in the genetic background of a *trpA* knockout mutant of L2, using the *trpA* gene from L2 under the control of a constitutive promoter. Whilst this shows that TrpA is an essential part of a properly functioning tryptophan synthase, the aggressive LGV serovars are more tolerant to tryptophan limitation than urogenital isolates (Caldwell et al., 2003), do not have an epithelial tropism and have no role in trachoma. Therefore, the biologically relevant experiment needed to complete the description of the indole rescue phenotype is the complementation of an ocular strain of *C. trachomatis* with the *trpRBA* operon of a urogenital strain. We chose to use the whole tryptophan synthase operon, including the native promoter (which is highly conserved among all serovars, as revealed by gene alignment), as this is more biologically relevant than using an artificial promoter. We hypothesised that complementation of the non-functional ocular *trpRBA* operon with the urogenital *trpRBA* operon would result in transfer of the indole rescuable phenotype from the urogenital donor to the ocular recipient strain.

Until now, only one study has documented the successful transformation of an ocular strain of *C. trachomatis* (Song et al., 2014), with the vast majority using L2 as the strain of choice in transformation experiments. This reliance on L2 as a recipient host results largely from restrictions on transformation of urogenital *C. trachomatis* isolates, which have recently been relaxed (National Institute of Health (NIH) Office of Science Policy (OSP) ruling of August 26th 2016). There is also a perceived need to tailor the transformation vector to the intended strain (Song et al., 2014). Transformation tropism has been described for some strains of *C. trachomatis*, for which there is apparently a strict requirement for a homologous plasmid to ensure transformation success (Song et al., 2014), although the study was unable to differentiate between transformation frequency (ability to get DNA into the EB) and barrier to replication (tropism). These observations do not apply to shuttle vector pSW2::GFP, which, despite having a urogenital chlamydia plasmid backbone, is capable of transforming LGV strain L2P- (Wang et al., 2013a). Until now, this has not been demonstrated in an ocular isolate.

We chose *C. trachomatis* strain A2497 as the first potential ocular recipient of pSW2::GFP, as this is a well-characterised strain [in that the genome sequence was available (Kari et al., 2011b), and the developmental cycle has been previously characterised (Porcella et al., 2015)]. Given the recent description of a genetic tropism barrier (Song et al., 2014), we initially chose to transform the plasmid-bearing wild type A2497 as it was previously found that recombination of a ~1 Kb region containing plasmid gene CDS2 facilitated the production of stable transformants when the closely related species *Chlamydia muridarum* was transformed with pSW2::GFP (Wang et al., 2014). This showed that in *C. muridarum* CDS2 is the minimal species-specific sequence for maintenance of the plasmid. We successfully recovered A2497 transformants but upon sequencing of the plasmid, it was clear that no recombination had occurred between the transforming vector and the native plasmid, proving that pSW2::GFP is compatible with the ocular *C. trachomatis* A2497 genome, and that CDS2 is not involved in plasmid tropism in *C. trachomatis*.

As recombination did not occur during transformation, we reasoned that pSW2::GFP should be capable of transforming plasmid-cured A2497. To test this notion, we cured A2497/pSW2::GFP of the shuttle vector so that we could re-transform the plasmid-cured A2497 with pSW2::GFP–the loss of GFP correlated with the loss of the pSW2::GFP vector, making the isolation of plasmid-free inclusions relatively straight forward in comparison to curing wild type A2497. The subsequent transformation of A2497P- was successful, yielding transformants that were morphologically indistinguishable from A2497/pSW2::GFP. Therefore, we now know that pSW2::GFP is capable of transforming *C. trachomatis* strains from the phylogenetically distant LGV strain L2, as well as more closely related urogenital and ocular biotypes, without the need to recombine with the native plasmid. This confirms the enhanced promiscuity of the pSW2::GFP plasmid, suggesting that it can be used for any strain of *C. trachomatis*.

Reasons for this enhanced strain compatibility are currently unknown. Analysis of 157 whole genome sequences showed that there was a strong association between chromosome genotype and plasmid, but that natural horizontal transfer of plasmids from ocular to urogenital strains has occurred (Versteeg et al., 2018). This occasional natural exchange of plasmids suggests that there is no cross-strain barrier to plasmid replication within the trachoma lineage, although this has not yet been reported between LGV and trachoma biovars. However, we know that at least in the case of vector pSW2::GFP, cross-biovar transformation is possible (Wang et al., 2013a). pSW2::GFP is derived from the plasmid of the Swedish Variant SW2 (Soderblom et al., 2006), which is the most divergent of the *C. trachomatis* plasmids, having a 377bp deletion in CDS1, and a 44 bp repeat upstream of CDS 2 (Ripa and Nilsson, 2006, 2007). These mutations have no effect on biological fitness of this strain (Unemo et al., 2010), but as the 44 bp repeat contains transcriptional start points for CDS2 and CDS3, it has been suggested that this duplication may have a role in expanding the host-range of pSW2 (Song et al., 2014; Wang et al., 2014).

pSW2::GFP is 4.5Kb larger than the native chlamydial plasmid and constitutively expresses a GFP-CAT fusion protein as well as β-lactamase (Wang et al., 2011a). We hypothesised that this might incur a fitness burden on transformed chlamydia; any reduction in fitness could compromise future transformation experiments–particularly given the low transformation efficiency of the system. Therefore, we performed one-step growth curves to compare the growth kinetics of the transformed strain to the wild type and plasmid-cured A2497. Maintenance of the plasmid was monitored throughout the developmental cycle by the observation of plasmid-containing inclusion morphology and green fluorescence under UV. Titrations of the chlamydia harvested at each of the 12 time-points revealed that there was no reduction in growth rate of the pSW2::GFP transformed strain in comparison to the wild type or plasmid-cured strains, proving that this plasmid is not a burden on the growth of A2497 (Figure 3A). The incoming shuttle vector eliminates the native plasmid (Wang et al., 2011a), so the actual increase in amount of replicating DNA is very small in comparison to the total bacterial DNA, especially given that the chlamydial plasmid is maintained at a low copy number (Pickett et al., 2005). Furthermore, the effect of plasmid carriage on bacterial fitness is complex (as reviewed by San Millan and MacLean San Millan and MacLean, 2017). Compensatory mechanisms may exist that alleviate any effect from carriage of the native plasmid (Bouma and Lenski, 1988; Harrison and Brockhurst, 2012) and increasing the size of this plasmid by inserting additional genes such as RSGFPCAT and β-lactamase may not be sufficiently burdensome to overcome these mechanisms. Together these factors may explain why pSW2::GFP carriage did not adversely affect the growth kinetics of A2497.

For delivery of the *trpRBA* operon, we chose pCDS5KO, which is derived from the pSW2::GFP shuttle vector, but with CDS5 deleted (Wang et al., 2013b). Our aim was to reduce the overall size of the plasmid vector–although we know that pSW2::GFP does not burden A2497, we wished to compensate for the addition of the 2.3Kb *trpRBA* operon by using this smaller shuttle vector. CDS5 encodes Pgp3, a key, secreted protein that has an important role in bacterial pathogenesis in mouse models of infection (Ramsey et al., 2014), but does not affect plasmid maintenance/stability (Wang et al., 2013b). Furthermore, we found that plasmid-free A2497 did not suffer any reduction in fitness in comparison to plasmid-carrying A2497 (wildtype or transformed), in accordance with previous reports of different plasmid-free strains (Peterson et al., 1990; Farencena et al., 1997). This supports the notion that using a plasmid without CDS5 in transformation experiments would have no effect on our *in vitro* experiments. Therefore, we considered the reduction in plasmid size justified using this derivative of the pSW2::GFP plasmid to enhance our probability of transformation success. Should these experiments be extended to animal models, the pSW2::GFP plasmid would be a more suitable vector for *trpRBA*; however, this is beyond the scope of the present study.

Soton D1 was chosen as the donor of the whole *trpRBA* operon as this isolate could be rescued from the effects of tryptophan starvation upon provision of indole, in line with its urogenital origin. The Soton D1 *trpRBA* operon is highly similar to that of A2497, bar the ocular strain-associated TrpA truncation and a single nonsynonymous polymorphism at nucleotide position 931 of the *trpB* gene (Supplementary Figure 3). This mutation is not one of the critical conserved residues identified as being necessary for TrpB activity (Fehlner-Gardiner et al., 2002). The *trpR* gene is highly conserved among all strains of *C. trachomatis* and is identical to that in A2497. Co-provision of the *trpR* gene ensured an appropriate level of repression in response to tryptophan presence, with the aim of mitigating any possible genome dosage effects caused by carriage of *trpRBA* on a multi-copy plasmid (additionally, plasmid copy number is known to be low in *C. trachomatis*; Pickett et al., 2005).

Upon growing A2497/pCDS5KO::trpRBA under tryptophan-depleted conditions, we showed that where ocular *C. trachomatis* strain A2497WT was not rescued upon provision of indole, A2497/pCDS5KO::trpRBA recovered to a similar titre as when grown in tryptophan-containing medium, proving that complementation by the urogenital *trpRBA* operon does indeed bestow an indole-rescuable phenotype on ocular chlamydia. There was some growth of Soton D1 and A2497/pCDS5KO::trpRBA in the absence of tryptophan or indole, which is not unprecedented (Fehlner-Gardiner et al., 2002; Caldwell et al., 2003; Mueller et al., 2016). This may be due to carry-over of inocula that did not infect during the first phase of the experiment, or low levels of tryptophan present in the inoculum. As varying sensitivities to tryptophan limitation have been described previously, with ocular strains being the most sensitive in comparison to urogenital and LGV strains (Caldwell et al., 2003), it seems that the residual tryptophan in these experiments may be enough to sustain some growth of Soton D1 and A2497/pCDS5KO::trpRBA but not A2497WT.

These data confirm that the *trpRBA* operon confers the ability to synthesise tryptophan from exogenous indole, in agreement with previous studies supporting the role of the active tryptophan synthase in urogenital tissue tropism (Fehlner-Gardiner et al., 2002; Caldwell et al., 2003; Kari et al., 2011a; Mueller et al., 2016). A recent study has identified an additional immune evasion tactic employed by *C. trachomatis* that blocks the host cell's response to IFN-γ. As a result only (uninfected) bystander cells can respond to IFN-γ, preventing the spread of the infection beyond the initially infected cell (Ibana et al., 2018). Timing appears to be critical as the authors found that pre-treatment of cells with IFN-γ prevents progression of *C. trachomatis* along the normal developmental cycle, whereas an existing infection will block IDO-1 expression. The ability to block IDO-1 may be preferable, as *de novo* synthesis of tryptophan is energetically expensive (Akashi and Gojobori, 2002). Furthermore, in the eye indole producers are exceedingly rare (or absent) and as classical trachoma strains do not have a functional tryptophan synthase, possession of an alternative mechanism of immune evasion may be advantageous.

Through the genetic complementation experiments conducted during the present study, we unequivocally confirm that the tryptophan operon is necessary and sufficient for the indole-rescue response of urogenital *C. trachomatis in vitro*, and that transfer of this operon to ocular strain A2497 restores the tryptophan biosynthesis capacity apparently lost during the niche adaptation process. In doing so we also show for the first time that pSW2::GFP derived shuttle vectors are compatible with the ocular *C. trachomatis* strain A2497 genome, building on the prior knowledge of its enhanced promiscuity and suggesting it as a universal transformation vector for *C. trachomatis* experiments.

Understanding tissue tropism in an obligate intracellular pathogen such as *C. trachomatis* is vital in unravelling the progress of chronic diseases such as trachoma, and may offer new insights leading to novel drug development and/or infection control strategies.

## Author Contributions

CO and IC conceptualised the study and designed all experiments; CO, PA, RS, SF, and SP performed experiments; CO wrote the manuscript; CO, IC, PA, RS, SF, and SP proof-read and edited the manuscript.

### Conflict of Interest Statement

The authors declare that the research was conducted in the absence of any commercial or financial relationships that could be construed as a potential conflict of interest.

## Abbreviations

EB: elementary body
RB: reticulate body
LGV: lymphogranuloma venereum
PID: pelvic inflammatory disease.
