# Supplementary material for: Genetic Transformation of a C. trachomatis Ocular Isolate With the Functional Tryptophan Synthase Operon Confers an Indole-Rescuable Phenotype
Source: Front Cell Infect Microbiol. 2018 Dec 14;8:434. doi: 10.3389/fcimb.2018.00434 (PMC6302012; doi:10.3389/fcimb.2018.00434)
Supplement: Supplementary file 1 [file Data_Sheet_1.PDF]

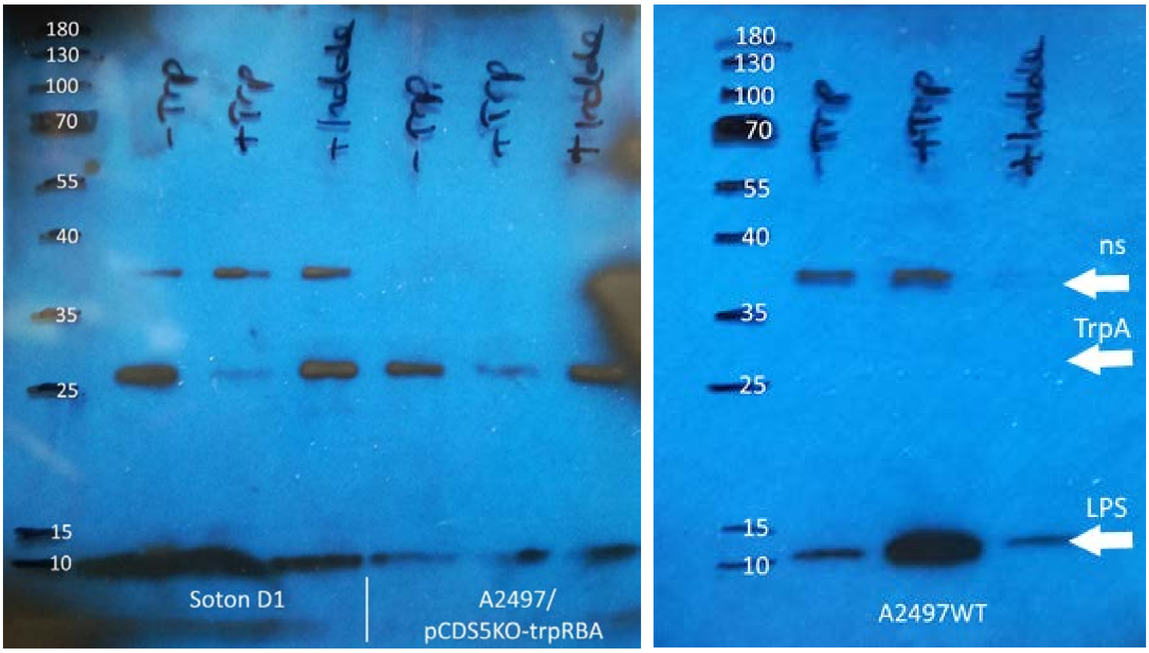

Supplementary Figure 1. Western blot analysis of TrpA expression using polyclonal anti-TrpA antisera. Strong TrpA expression (at around 27 KDa) was seen from the -Trp and +Indole wells for Soton D1 and A2497/pCDS5KO::trpRBA, but no discernible TrpA expression was seen for A2497WT. The lack of detection of a TrpA fragment (around 7KDa) from A2497WT may be explained by the loss of antigenic epitopes in this greatly truncated form of the protein. Loading controls using anti-LPS monoclonal antibody MAb29 were also performed to indicate the chlamydia growth yield under each growth condition. For strains D and A2497/pCDS5KO::trpRBA there was not much difference in yield between each of the growth conditions, whereas there was much more growth in the +Trp well for A2497WT in comparison to the -Trp and +Indole wells. This is expected as A2497 cannot synthesise tryptophan due to the absence of an intact TrpA and thus growth was inhibited in the absence of tryptophan in the medium. There is also a non-specific protein (labelled as “ns”) at around 38KDa in all strains (although very faint in A2497/pCDS5KO::trpRBA). These data show that TrpA is being expressed by A2497/pCDS5KO::trpRBA. Secondly, the expression in the +Trp medium appears to be lower for both D and A2497/pCDS5KO::trpRBA, which may suggest repression of the *trpRBA* operon by TrpR due to the presence of excess tryptophan.
