# Supplementary material for: Genetic Transformation of a C. trachomatis Ocular Isolate With the Functional Tryptophan Synthase Operon Confers an Indole-Rescuable Phenotype
Source: Front Cell Infect Microbiol. 2018 Dec 14;8:434. doi: 10.3389/fcimb.2018.00434 (PMC6302012; doi:10.3389/fcimb.2018.00434)
Supplement: Supplementary file 2 [file Data_Sheet_2.PDF]

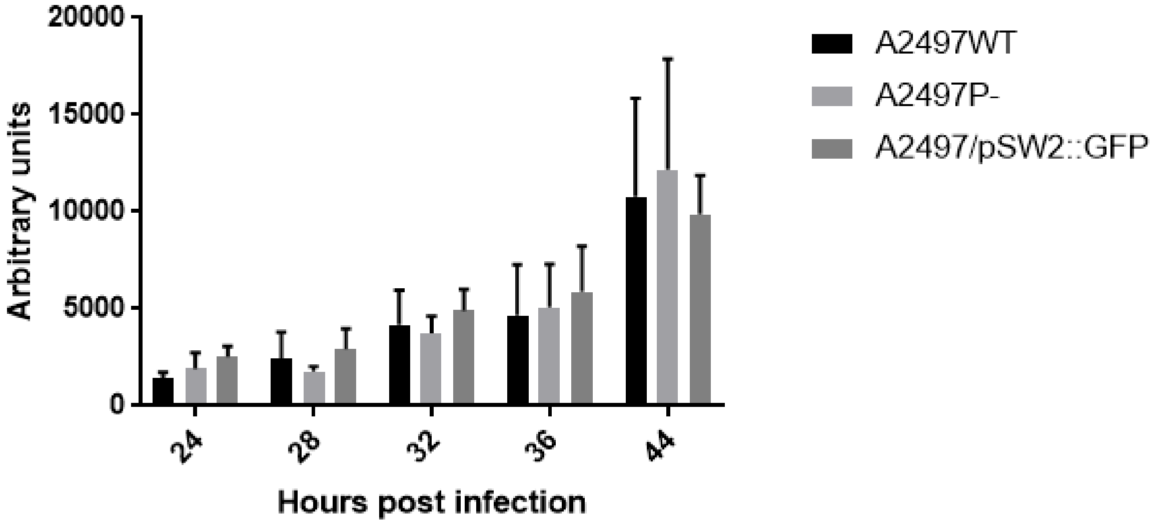

Supplementary Figure 2. Inclusion size throughout the growth cycle. Confocal images (as shown in Figure 3) were taken at the stated time points. The areas of inclusions in the images were measured using ImageJ software and are presented in arbitrary units of area calculated by the software. A two-way Anova (n=6) was done using GraphPad Prism, showing that there was no significant difference in inclusion size between the three strains at any time point.
