# Supplementary material for: Genetic Transformation of a C. trachomatis Ocular Isolate With the Functional Tryptophan Synthase Operon Confers an Indole-Rescuable Phenotype
Source: Front Cell Infect Microbiol. 2018 Dec 14;8:434. doi: 10.3389/fcimb.2018.00434 (PMC6302012; doi:10.3389/fcimb.2018.00434)
Supplement: Supplementary file 3 [file Data_Sheet_3.PDF]

Alignment Report of 'TrpRBA: SotonD1 vs A2497' - ClustalW (Weighted)

HE601798.1:192800-195486_SotonD1 CGTGATCCTTATTCCTAGAGGGGAGAATTCTAAGAAAAGAAAATAATGTAGCATATATTTATGAAATGTTGTAATATTAT 80

FM872306.1:194316-196981_A2497 CGTGATCCTTATTCCTAGAGGGGAGAATTCTAAGAAAAGAAAATAATGTAGCATATATTTATGAAATGTTGTAATATTAT 80

HE601798.1:192800-195486_SotonD1 AGCATTACAAAAAGGTGCGATATGAAAAATCAAGAGGAGTCTGGCTGGCAAGCTTTTCTGACATTATGCTCTAAAATGCA 160

FM872306.1:194316-196981_A2497 AGCATTACAAAAAGGTGCGATATGAAAAATCAAGAGGAGTCTGGCTGGCAAGCTTTTCTGACATTATGCTCTAAAATGCA 160

HE601798.1:192800-195486_SotonD1 AAAAGAAAAGTTTTTACAAGACCTTTTTTCGCTGTTTTTGTCTTTTAGCGAACGTAAAGATGTCGCTTCTCGCTATCATA 240

FM872306.1:194316-196981_A2497 AAAAGAAAAGTTTTTACAAGACCTTTTTTCGCTGTTTTTGTCTTTTAGCGAACGTAAAGATGTCGCTTCTCGCTATCATA 240

HE601798.1:192800-195486_SotonD1 TCATTCGAGCTCTTTTAGAAGGGGAGCTCACTCAAAGAGAGATAGCAGAGAAATACGGAGTCAGTATCGCACAAATTACC 320

FM872306.1:194316-196981_A2497 TCATTCGAGCTCTTTTAGAAGGGGAGCTCACTCAAAGAGAGATAGCAGAGAAATACGGAGTCAGTATCGCACAAATTACC 320

HE601798.1:192800-195486_SotonD1 AGAGGATCTAATGCCCTTAAAGGATTAGATCCTCAATTTAAAGAGTTTTTACAAAAAGAGATCTGATCTTCTTTTGTAAA 400

FM872306.1:194316-196981_A2497 AGAGGATCTAATGCCCTTAAAGGATTAGATCCTCAATTTAAAGAGTTTTTACAAAAAGAGATCTGATCTTCTTTTGTAAA 400

HE601798.1:192800-195486_SotonD1 ATACAAATAAGATTAAAAGTATTTGTATGCATGCGTTGTTAATGAACAAATATTCTGTTTTAGCAGTTTTGGTACATAAG 480

FM872306.1:194316-196981_A2497 ATACAAATAAGATTAAAAGTATTTGTATGCATGCGTTGTTAATGAACAAATATTCTGTTTTAGCAGTTTTGGTACATAAG 480

HE601798.1:192800-195486_SotonD1 TATAGCTGCAGCATGCCATGCAAATCAGCTTTTCAAGCTGATTGCTTCCAAGATATTCAAAAATTCATCCTCTTACAGCG 560

FM872306.1:194316-196981_A2497 TATAGCTGCAGCATGCCATGCAAATCAGCTTTTCAAGCTGATTGCTTCCAAGATATTCAAAAATTCATCCTCTTACAGCG 560

HE601798.1:192800-195486_SotonD1 TGCCTGGCTTTCTTTTGAAAGCTGGCGCTTATCTACTTGGCGATAGGCCTAATTAAGAAGCCTTTTATTTGATTAAGAGA 640

FM872306.1:194316-196981_A2497 TGCCTGGCTTTCTTTTGAAAGCTGGCGCTTATCTACTTGGCGATAGGCCTAATTAAGAAGCCTTTTATTTGATTAAGAGA 640

HE601798.1:192800-195486_SotonD1 TGTTCTTATAGAAGTAAGAGCGTCTTTTTTGCGCAGGATTATTCTGTCGCCAGTTTTTTCTATGATTTTAACACTATAAT 720

FM872306.1:194316-196981_A2497 TGTTCTTATAGAAGTAAGAGCGTCTTTTTTGCGCAGGATTATTCTGTCGCCAGTTTTTTCTATGATTTTAACACTATAAT 720

HE601798.1:192800-195486_SotonD1 TTTATGGAGAAAAGATGTTCAAACATAAACATCCTTTTGGGGGAGCGTTCCTTCCCGAAGAACTATTAGCCCCTATACAG 800

FM872306.1:194316-196981_A2497 TTTATGGAGAAAAGATGTTCAAACATAAACATCCTTTTGGGGGAGCGTTCCTTCCCGAAGAACTATTAGCCCCTATACAG 800

HE601798.1:192800-195486_SotonD1 AATCTAAAAGCGGAATGGGAGATTCTCAAAACTCAGCAAAGTTTTTTATCTGAACTAGATTGTATTTTGAAAAACTATGC 880

FM872306.1:194316-196981_A2497 AATCTAAAAGCGGAATGGGAGATTCTCAAAACTCAGCAAAGTTTTTTATCTGAACTAGATTGTATTTTGAAAAACTATGC 880

HE601798.1:192800-195486_SotonD1 GGGGAGACAAACTCCTCTGACTGAAGTTAAGAATTTTGCTCGAGCTATTGATGGCCCTAGAGTATTTCTTAAACGCGAAG 960

FM872306.1:194316-196981_A2497 GGGGAGACAAACTCCTCTGACTGAAGTTAAGAATTTTGCTCGAGCTATTGATGGCCCTAGAGTATTTCTTAAACGCGAAG 960

HE601798.1:192800-195486_SotonD1 ATCTTTTGCATACAGGAGCACATAAACTGAATAATGCTCTTGGTCAGTGTTTGCTTGCTAAATATCTTGGGAAAACACGT 1040

FM872306.1:194316-196981_A2497 ATCTTTTGCATACAGGAGCACATAAACTGAATAATGCTCTTGGTCAGTGTTTGCTTGCTAAATATCTTGGGAAAACACGT 1040

HE601798.1:192800-195486_SotonD1 GTTGTAGCTGAAACAGGTGCGGGACAACATGGAGTAGCAACAGCAACAGCGTGTGCTTATCTAGGATTAGATTGTGTAGT 1120

FM872306.1:194316-196981_A2497 GTTGTAGCTGAAACAGGTGCGGGACAACATGGAGTAGCAACAGCAACAGCGTGTGCTTATCTAGGATTAGATTGTGTAGT 1120

HE601798.1:192800-195486_SotonD1 ATACATGGGAGCAAAAGATGTGGAACGACAGAAACCAAATGTAGAGAAAATGCGCTTTTTAGGTGCTGAAGTCGTTTCTG 1200

FM872306.1:194316-196981_A2497 ATACATGGGAGCAAAAGATGTGGAACGACAGAAACCAAATGTAGAGAAAATGCGCTTTTTAGGTGCTGAAGTCGTTTCTG 1200

HE601798.1:192800-195486_SotonD1 TAACAAAAGGATCTTGTGGACTCAAAGATGCAGTTAATCAAGCTCTACAAGATTGGGCAACAACACACTCATTTACTCAC 1280

FM872306.1:194316-196981_A2497 TAACAAAAGGATCTTGTGGACTCAAAGATGCAGTTAATCAAGCTCTACAAGATTGGGCAACAACACACTCATTTACTCAC 1280

HE601798.1:192800-195486_SotonD1 TATTGCTTAGGATCGGCCTTAGGACCTTTACCTTATCCCGATATCGTTCGATTTTTTCAGTCTGTTATAAGCGCTGAAGT 1360

FM872306.1:194316-196981_A2497 TATTGCTTAGGATCGGCCTTAGGACCTTTACCTTATCCCGATATCGTTCGATTTTTTCAGTCTGTTATAAGCGCTGAAGT 1360

HE601798.1:192800-195486_SotonD1 GAAAGAGCAAATCCATGCAGTTGCAGGAAGAGATCCTGATATTCTGATTGCATGTATCGGAGGTGGCTCCAACGCTATTG 1440

FM872306.1:194316-196981_A2497 GAAAGAGCAAATCCATGCAGTTGCAGGAAGAGATCCTGATATTCTGATTGCATGTATCGGAGGTGGCTCCAACGCTATTG 1440

HE601798.1:192800-195486_SotonD1 GATTTTTCCATCATTTTATCCCGAATCCAAAAGTCCAATTAATTGGAGTGGAAGGGGGAGGACTGGGCATTTCTTCAGGA 1520

FM872306.1:194316-196981_A2497 GATTTTTCCATCATTTTATCCCGAATCCAAAAGTCCAATTAATTGGAGTGGAAGGGGGAGGACTGGGCATTTCTTCAGGA 1520

HE601798.1:192800-195486_SotonD1 AAACATGCAGCACGTTTTGCAACAGGGCGACCTGGAGTATTCCACGGATTTTATTCGTATCTTCTTCAAGATGACGATGG 1600

FM872306.1:194316-196981_A2497 AAACATGCAGCACGTTTTGCAACAGGGCGACCTGGAGTATTCCACGGATTTTATTCGTATCTTCTTCAAGATGACGATGG 1600

HE601798.1:192800-195486_SotonD1 ACAAGTATTACAAACTCACTCCATTTCCGCTGGATTAGATTATCCTTCAGTTGGGCCAGATCATGCCGAAATGCATGAGT 1680

FM872306.1:194316-196981_A2497 ACAAGTATTACAAACTCACTCCATTTCCGCTGGATTAGATTATCCTTCAGTTGGGCCAGATCATACCGAAATGCATGAGT 1680

HE601798.1:192800-195486_SotonD1 CAGGACGAGCCTTTTATACATTAGCCACCGATGAAGAGGCGTTACGAGCTTTTTTCCTGCTTACTAGAAACGAGGGGATT 1760

FM872306.1:194316-196981_A2497 CAGGACGAGCCTTTTATACATTAGCCACCGATGAAGAGGCGTTACGAGCTTTTTTCCTGCTTACTAGAAACGAGGGGATT 1760

HE601798.1:192800-195486_SotonD1 ATTCCTGCATTGGAGTCTTCACATGCTCTCGCACATTTAGTTTCGATTGCTCCTTCTCTACCAAAGGAACAAATCGTCAT 1840

FM872306.1:194316-196981_A2497 ATTCCTGCATTGGAGTCTTCACATGCTCTCGCACATTTAGTTTCGATTGCTCCTTCTCTACCAAAGGAACAAATCGTCAT 1840

HE601798.1:192800-195486_SotonD1 CGTTAACTTATCTGGAAGAGGTGATAAGGATCTTCCACAAATCATCCGCAGAAACAGAGGAATTTATGAGTAAATTAACC 1920

FM872306.1:194316-196981_A2497 CGTTAACTTATCTGGAAGAGGTGATAAGGATCTTCCACAAATCATCCGCAGAAACAGAGGAATTTATGAGTAAATTAACC 1920

HE601798.1:192800-195486_SotonD1 CAAGTTTTTAAACAAACTAAGCCATGTATTGGCTATCTAACCGCTGGTGATGGCGGTACTAGTTATACTATTGAGGCGGC 2000

FM872306.1:194316-196981_A2497 CAAGTTTTTAAACAAACTAAGCCATGTATTGGCTATCTAACCGCTGGTGATGGCGGTACTAGTTATACTATTGAGGCGGC 2000

HE601798.1:192800-195486_SotonD1 AAAAGCTCTGATTCAAGGAGGTGT- - CGATATTCTGGAACTAGGATTTCCTTTTTCTGATCCTGTTGCAGATAATCCAGA 2080

FM872306.1:194316-196981_A2497 AAAAGCTCTGATTCAAGGAGGTGTGTCGATATTCTGGAACTAGGATTTCCTTTTTCTGATCCTGTTGCAGATAATCCAGA 2080

HE601798.1:192800-195486_SotonD1 AATTCAAGTATCTCATGATCGGGCTTTAGCAGAAAATCTGACGTCAGAAACTTTGTTAGAGATCGTAGAAGGTATCCGAG 2160

FM872306.1:194316-196981_A2497 AATTCAAGTATCTCATGATCGGGCTTTAGCAGAAAATCTGACGTCAGAAACTTTGTTAGAGATCGTAGAAGGTATCCGAG 2160

HE601798.1:192800-195486_SotonD1 CTTTTAATCAAGAAGTCCCATTGATCTTATATAGCTACTACAATCCGCTTCTACAAAGGGACTTAGATTATCTACGCAGA 2240

FM872306.1:194316-196981_A2497 CTTTTAATCAAGAAGTCCCATTGATCTTATATAGCTACTACAATCCGCTTCTACAAAGGGACTTAGATTATCTACGCAGA 2240

HE601798.1:192800-195486_SotonD1 CTAAAAGACGCGGGAATAAATGGTGTGTGCGTTATAGATCTTCCAGCACCTTTATCACACGGAGAAAAATCTCCATTTTT 2320

FM872306.1:194316-196981_A2497 CTAAAAGACGCGGGAATAAATGGTGTGTGCGTTATAGATCTTCCAGCACCTTTATCACACGGAGAAAAATCTCC - - -TTT 2320

HE601798.1:192800-195486_SotonD1 TGAAGATCTTTTAGCTGTAGGATTGGATCCTATTTTGCTTATTTCTGCAGGGACAACGCCGGAGCGGATGTCTTTAATAC 2400

FM872306.1:194316-196981_A2497 TGAAGATCTTTTAGCTGTAGGATTGGATCCTATTTTGCTTATTTCTGCAGGGACAACTCCGGAGCGGATGTCTTTAATAC 2400

HE601798.1:192800-195486_SotonD1 AAGAATACGCAAGAGGCTTTCTGTATTATATCCCATATCAAGCTACGAGAGATTCTGAAGTAGGTATCAAAGAAGAATTT 2480

FM872306.1:194316-196981_A2497 AAGAACACGCAAGAGGCCTTCTGTATTATATCCCATACCAAGCTACGAGAGATTCTGAAGTAGGTATCAAAGAAGAATTT 2480

HE601798.1:192800-195486_SotonD1 CGAAAAGTCAGAGAACATTTTGATCTTCCAATTGTAGATAGAAGAGATATTTGTGATAAAAAAGAAGCTGCACATGTGCT 2560

FM872306.1:194316-196981_A2497 CGAAAAGTCAGAGAACATTTTGATCTTCCAATTGTAGATAGAAGAGATATTTGTGATAAAAAAGAAGCTGCACATGTGCT 2560

HE601798.1:192800-195486_SotonD1	GAATTATTCAGATGGTTTCATTGTGAAAACAGCGTTTGTTCATCAGACAACAATGGATTCTTCGGTAGAGACTCTGACTG	2640
FM872306.1:194316-196981_A2497	GAATTATTCAGATGGTTTCATTGTGAAAACAGCGTTTGTTCATCAGACAACAATGGATTCTTCGGTAGAGACTCTGACTG	2640
HE601798.1:192800-195486_SotonD1	CACTTGCACAAACAGTTATTCCTGGATAA	2669
FM872306.1:194316-196981_A2497	CACTTGCACAAACAGTTATTCCTGGATAA	2669

Gene boundaries:

1-46: End of upstream gene CTO_0181 (adherance factor)

102-386: trpR

427-606: hypothetical protein

735-1913: trpB

1906-2666: trpA

Key:

Variable sites are underlined.

Deletions are marked with a hyphen (-).
